# Towards Healthy Aging through Semantic Enrichment

**Published:** 2026-03-02

**Authors:** John Beverley, Julie C. Bowker, Hollen N. Reischer, Rachel A. Mavrovich, Regina Hurley, Sean Kindya, Sam Smith, Jie Zheng, Yongqun He, Damayanthi Beera, William D. Duncan

**Affiliations:** 1 Department of Philosophy, University at Buffalo, Buffalo NY, USA; 2 National Center for Ontological Research, Buffalo NY, USA; 3 Institute for Artificial Intelligence and Data Science, Buffalo NY, USA; 4 Department of Psychology, University at Buffalo, Buffalo NY, USA; 5 University of Michigan Medical School, Ann Arbor, MI, USA; 6 College of Dentistry, University of Florida, Gainesville, FL, USA

**Keywords:** Ontology Engineering, Gerotranscendence, Solitude, Basic Formal Ontology, Healthy Aging

## Abstract

This paper introduces two interoperable ontologies—SOLO (Solitude Ontology) and GERO (Gerotranscendence Ontology)—developed to formalize key psychological constructs relevant to healthy aging. Grounded in the Behavioral Change Intervention Ontology, the ontologies clarify distinctions between terms such as solitude, loneliness, self-transcendence, and gerotranscendence, and encode their realization conditions across the lifespan. By integrating validated psychometric instruments and supporting structured responses to competency questions, the ontologies enable semantic reasoning, cross-disciplinary data integration, and development of ontology-driven tools for aging research and intervention design.

## 1. Introduction

A substantial minority (25%) of U.S. adults 65 years old or older live alone and feel socially isolated [1]. This is a clear public health problem due to significant links between social isolation and a myriad of physical and psychological difficulties, including early mortality [2]. It has been suggested that younger and middle-aged adults spend 35% of their time alone [3], in contrast to older adults for whom this increases to 70% [4]. Time alone increases during older age due to changing needs and priorities in how and with whom one spends their time as well as social role and relationship losses [5, 6]. As researchers have learned, however, being alone may reflect a variety of psychological constructs, e.g. solitude [7, 8], social isolation [9], and loneliness [10]. Clarifying subtleties of these constructs is crucial for exploring impacts on well-being, especially given that studies of one construct often use assessments of another, e.g. studies of loneliness will use measures descriptive of social isolation [11].

Although rarely considered together, there seem to be clear relationships between solitude and gerotranscendence. Gerotranscendence is a psychological phenomenon occurring in older adulthood that is associated with shifts in goals, values, and perspectives towards greater feelings of connection to past and future generations [12], attention to personal meaning [13], and seeking of coherence in one’s life story [14]. Researchers in developmental, personality, and clinical psychology, as well as in nursing and other health sciences, investigate constructs such as gerotranscendence, self-transcendence, ego integrity, and sense of coherence, using a range of theories and measurement approaches and definitions. The lack of standardized meanings for gerotranscendence constructs has resulted in a body of research that demonstrates a compelling, but disjointed, story about the role of gerotranscendence in promoting healthy aging, or robust physical, mental, and cognitive health in older adulthood.

Ambiguities within each literature amplify disconnects between them. For example, longitudinal studies of solitude in old age are uncommon [15], but are more common in gerotranscendence studies [16]. Yet, social relationships change over the lifespan and plausibly influence experiences of solitude and feelings of connectedness beyond oneself [17]. Similarly, most of the published work on solitude across the lifespan has focused on negative outcomes, unlike the work on gerotranscendence, which has been more balanced in its focus on both positive and negative outcomes. Moreover, momentary experiences of solitude have been investigated by solitude researchers [1, 18], while gerotranscendence studies typically focus on longer-term experiences.

**Figure 1:**
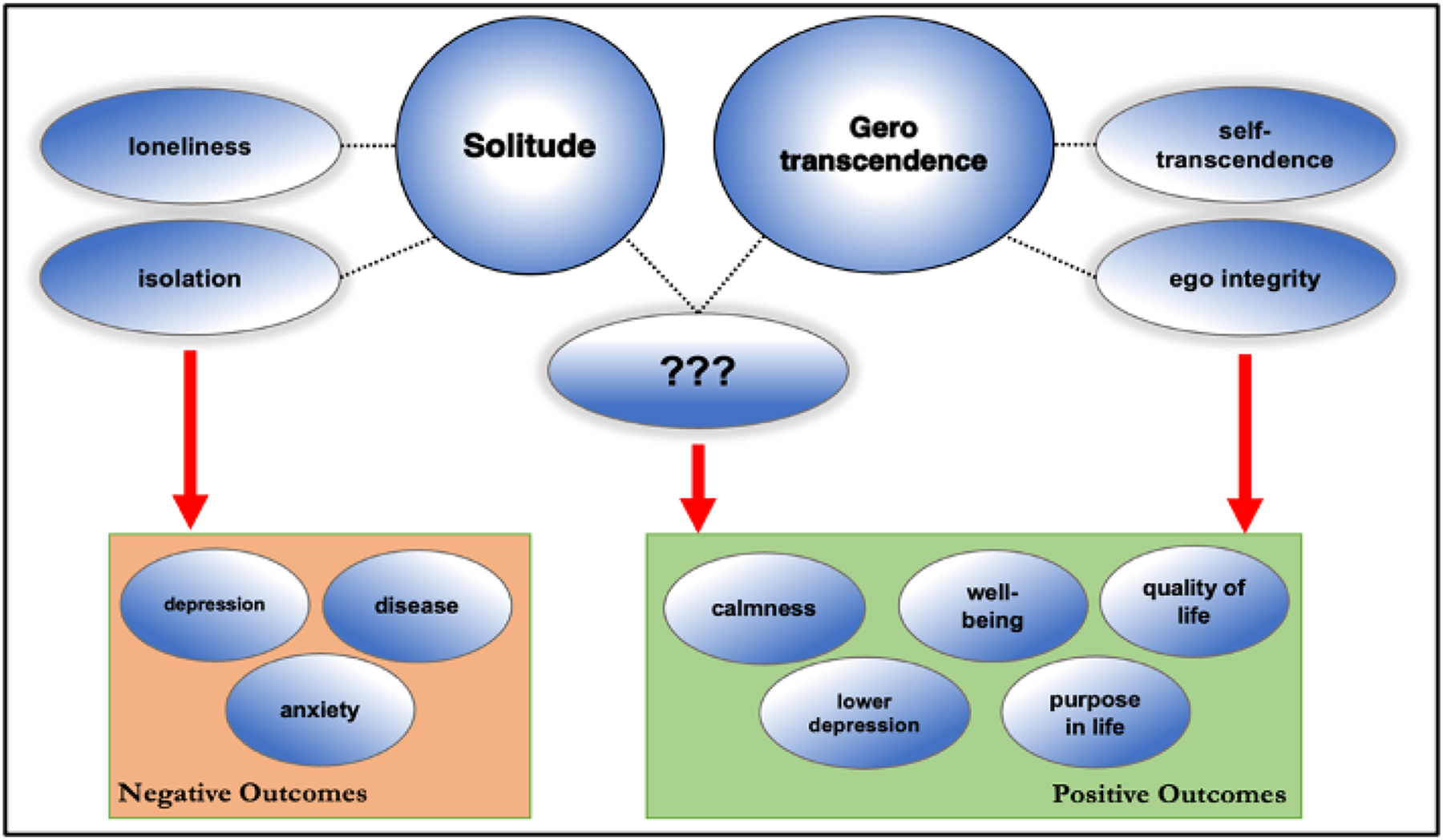
Solitude and Gerotranscendence Relationships in Older Adults, and Potential Overlap

Despite these differences, it is not difficult to identify where these literatures may connect. For example, solitude researchers have explicitly measured behaviors and attitudes related to solitude as indicators of gerotranscendence, e.g. “I am more likely to engage in quiet contemplation”, “I am less interested in superficial social contacts.” Additionally, experiences with social isolation and loneliness are sometimes construed as indicating a lack of gerotranscendence, e.g. “I feel more isolated and lonely.” [12, 19] Solitude researchers are becoming interested in different motivations for, and positive versus negative beliefs about, solitude. There is evidence that preferences for and positive feelings about solitude help to explain which older aged individuals may flourish in solitude. This focus of research, however, has developed in isolation, despite connections to gerotranscendence literature. We maintain that viewing solitude in older adulthood through relationships to gerotranscendence will illuminate connections to positive outcomes (Figure 1) and help explain not only which older adults, but also why some older adults, are able to experience psychological benefits from being alone.

To clarify these relationships, we leverage ontologies – logically defined controlled vocabularies of terms and logical relationships among them [20] – to represent gerotranscendence and solitude constructs, theories, measurements, and associated empirical research. In doing so, we aim to provide a machine-readable, formally rigorous, foundation from which researchers will be able to aggregate solitude and gerotranscendence data, identify research gaps, and leverage existing data in the interest of promoting healthy aging [21].

In what follows, we outline the results of our ontological investigation of these domains. After outlining our ontology development methodology, we describe the Gerotranscendence Ontology (GERO) and the Solitude Ontology (SOLO).^1^ Various choice points are defended and design patterns introduced. We close by highlighting applications of this work, limitations, and future work, such as the deployment of our ontology to generative AI agents to be used by researchers investigating how solitude relates to flourishing in older age.

## 2. Methods

Development of GERO and SOLO followed ontology engineering best practices, leveraging existing standards and reusing domain-level ontology content where possible, extending from top- and mid-level ontologies, establishing a CI/CD development workflow, and employing competency questions generated by domain experts.

### 2.1. Ontology Development Framework

First GERO and SOLO are represented using the OWL 2 Web Ontology Language (OWL 2 DL), which is a decidable fragment of first-order logic, supporting automated reasoning, consistency checking, and classification using reasoners such as HermiT and Pellet [22]. Our ontologies abide by widely held principles for ontology design, development, maintenance, and implementation. Ontology elements are given global unique identifiers, natural language definitions, synonyms, editor attribution, and source references annotated using standards such as the Resource Description Framework (RDF), and Simple Knowledge Organization System (SKOS), and so on.

### 2.2. Hub-and-Spoke

We adopt Basic Formal Ontology (BFO) as a starting point for development [23]. BFO is an ISO 21838–2 top-level ontology standard [24] used in over 700 open-source projects, such as those found in the Open Biological and Biomedical Ontology (OBO) Foundry [25], the Industrial Ontologies Foundry (IOF) [26], and the Common Core Ontologies (CCO) [26]. Most relevant here, BFO serves as the foundation for the Behavior Change Intervention Ontology (BCIO) [27] which serves as the central spoke for our work. BCIO contains classes and relations representing content common to all behaviour intervention, e.g. **behaviour change technique**^2^ and **antisocial behaviour**. SOLO and GERO extend BCIO by introducing domain-specific classes, following a downward population strategy where new terms fall under leaf terms in the BCIO hierarchy. For example, the BCIO class **psychological need** provides a proper parent for the GERO class **need for a sense of belonging to a community**, defined as a psychological need to feel connected to and accepted by a group. Extending from BCIO helps avoid the creation of ontology silos [20] ontologies which share a domain but are not interoperable.

Where BCIO lacks domain coverage, we reused where possible terms and relations from existing BFO-conformant ontologies, such as the Ontology for Biomedical Investigations (OBI) [25], the Ontology of Medically Related Social Entities (OMRSE) [28], the Gene Ontology [29], the Mental Functioning Ontology [30], and the Ontology of Statistical Methods [31]. Such reuse promotes semantic interoperability across distinct but overlapping ontology efforts and so promotes easier integration of data enriched by ontologies. Where no well-developed terms or relations could be identified needed to represent domain entities, new novel ontology elements were introduced, after consultation with domain experts and relevant literature. Developers constructed logical definitions for terms and relations, guided by best practices for definition construction [20].

### 2.3. CI/CD Workflow

Our ontology development workflow was conducted on GitHub, which is used for version control, issue tracking, stakeholder feedback, and automated release workflows. Automated quality control was implemented leveraging ROBOT [32] and SPARQL, ensuring – among other things – that annotation conventions are adopted, import statements are correct, and common ontology modeling mistakes avoided. Development follows a secure CI/CD [33] model using GitHub Actions, which allow for running integration tests to ensure data consistency and integrity, deploying a release to stakeholders, and deployment of artifacts to a staging environment for review. Ontologies are validated before deployment using ROBOT report tests, and competency question queries. All artifacts are publicly released under an open license.

### 2.4. Stakeholder Centered Design

We engage a multidisciplinary group of stakeholders including researchers, clinicians, and behavioral scientists with domain expertise in solitude, gerotranscendence, psychology, and public health. Ontology development is guided by domain-specific Competency Questions (CQs) [34, 35]. CQs are used in ontology and knowledge graph development to delimit scope, focus design efforts, standardize documentation, and promote the accuracy of representations. Identification of content for a given domain is informed by and informs CQ development. Answering CQs helps facilitate identification of available experimental and theoretical support for a given construct and lowers the likelihood that researchers unknowingly replicate results or produce outdated research.

Table 1 displays sample CQs guiding the engineering of GERO and SOLO, provided by our domain experts. GERO and SOLO were designed to provide sufficient ontological content to answer such CQs, as demonstrated later in this overview. CQs and ontology content will ultimately be integrated into a platform with question-answer and recommender features, to help gerotranscendence and solitude researchers investigate their respective domains.^3^

## 3. GERO and SOLO

We seek to balance rigor and practicality by building consensus around content to be codified in two domain-level ontologies – GERO and SOLO – focused on constructs, behaviours, interventions, theories, and outcomes for respective domains. Table 2 displays selected reused ontology terms relevant to the discussion to follow. Additionally, this table displays newly introduced terms common to both GERO and SOLO, which we aim to either have added to BCIO or a reference ontology extending from it from which GERO and SOLO extend.^4^

### 3.1. Gerotranscendence Ontology (GERO)

Research suggests there are significant positive associations between gerotranscendence, well-being, and mental health, such as higher life satisfaction and lower levels of depression [36, 16]. However, as with solitude literature, it is not always clear how gerotranscendence results relate across studies. For example, nursing and psychology literatures both use “self-transcendence” to describe generally gerotranscendent phenomena, including increased wisdom, death acceptance, and meaning making. Yet only nursing measures typically examine attitudes towards community engagement, whereas only developmental psychology measures examine the role of love in transcendence [19].

Given the wide variety of terminology use in this domain, our primary goal is to represent as clearly as possible the many, sub-discipline, domain-expert, or perhaps even research article-specific, uses of terminology relevant to gerotranscendence. This will require, say, representing development psychology characterizations of gerotranscendence, as well as nursing senses, and so on. If we have represented terminological use in these areas accurately and researchers in these areas are using terminology the same way, and, then our ontologies will demonstrate convergence; otherwise, our ontologies will demonstrate divergence. In any event, to achieve our primary goal here we must first provide a semantic foundation upon which to represent as clearly as possible terminological ambiguity, which is our task in this section.

Ontologically, **transcendence** can be understood as a **mental disposition** involving a shift in perspective—away from immediate, self-centered goals and toward broader narratives that incorporate past and future generations. Such a shift in perspective often entails reorientation toward deeper meaning, coherence in one’s life story, or reconciliation with existential realities.

Within this broader genus, **self-transcendence** is a more specific species of **transcendence** characterized by a movement beyond the self toward moral, existential, or spiritual concerns. These include prosocial commitments such as care for others, engagement with the natural world, and alignment with universal values. **Self-transcendence** is not necessarily age-bound - it can be experienced across the lifespan - but it is often deepened by reflective processes that accompany aging. **Gerotranscendence**, on the other hand, is a type of **self-transcendence** that is realized specifically in older adults. It retains the same structural features - existential orientation, generativity, expanded moral concern - but is shaped by the aging process, and involves acceptance of death, reevaluation of time, and integration of one’s life narrative. In this way, **gerotranscendence** is not merely an increase in spirituality or social concern, but a patterned developmental shift characterized by cosmic perspective and interiority.

This developmental trajectory is partially anticipated in Erikson’s **ego integrity theory**, which identifies acceptance of one’s life as a central psychological task in late adulthood. Achieving ego integrity involves embracing both accomplishments and failures without regret and reaching a peaceful reconciliation with the inevitability of death. While ego integrity is not identical to **gerotranscendence**, it putatively provides a developmental and emotional foundation for it. Where ego integrity culminates in acceptance, **gerotranscendence** often expands that acceptance into connection—with history, future generations, nature, and the universe. Recognizing these distinctions is, moreover, critical for both measurement and intervention, such as the **Gerotranscendence Scale**.

Our initial definitions for high-value terms in the gerotranscendence literature will serve as a foundation from which we will derive others, such as developmental psychology-specific characterizations of gerotranscendence or self-transcendence. In that respect, we do not intend the definitions provided here to be univocal and universal. Rather, they are intended to reflect what is common across a wide range of ambiguous uses of these terms.

### 3.2. Solitude Ontology (SOLO)

As a construct, solitude is a state typically defined by physical aloneness or the absence of social interaction [7]; too much time in solitude has been associated with psychological distress across the lifespan, but some time in solitude has also been linked to increased calmness and lower rates of depression in older adults [8]. Social isolation may be described as an objective lack of social connection and has been linked to depression and increased mortality [8, 9]. Loneliness, on the other hand, is a subjective feeling of dissatisfaction with social relationships, similarly linked to depression and increased mortality, and increased cortisol levels [10, 11, 37]. These observations provide a scientifically informed foundation on which to construct SOLO terms. As with GERO, our task here is primarily to represent as clearly as possible the many, sub-discipline, domain-expert, or perhaps even research article-specific, uses of solitude terminology; our definitions to follow are intended to serve as a starting point from which to derive such other uses of such terminology.

Note that “state” talk in the characterizing of solitude is often ambiguous between **dispositions**, such as my **disposition** to laugh at a joke, or **processes**, such as my laughing at a joke. Given that solitude thus described is connected to what are arguably states in the **process** sense, we tie our ontological representation of solitude as a **disposition** to processes in which it may be realized, such as **being physically alone**^5^ or lacking social interaction. In other words, **being physically alone** is a **process** that may satisfy the first disjunct; **loneliness**^6^ is a **process** which is the realization of the latter disjunct. Repeated or prolonged realizations of these states may lead to the formation of a **social isolation state**, a **process** in which an individual objectively lacks meaningful social connection. When recurrent, such states may result in the internalization of a **social isolation disposition**, a **mental disposition** already recognized within BCIO.

**Social withdrawal** may evolve out of anxiety or trauma, as a habitual or fearful avoidance of social interactions, throughout the life span. That said, in late adulthood - a life stage characterized by transitions in identity, role, and social structure - individuals may develop a novel relationship to **solitude**. Specifically, **older adults** often demonstrate what we characterize as **solitude affinity**:^7^ a preference for and enjoyment of time spent alone, especially for the purposes of reflection, restoration, and creativity. Individuals may experience barriers to realizing solitude affinity. In such cases, where solitude is desired but unobtainable, individuals may experience **aloneliness** - a **disposition** realized in distress from a perceived lack of desired alone time. This concept provides a counterpoint to loneliness, reinforcing that well-being is affected not only by deficits in **social interaction** but also by deficits in **solitude**.

The **social convoy model** provides additional context for understanding the shifting dynamics of social relationships over time. This model suggests that social connections are structured in concentric circles of closeness, with peripheral ties being more susceptible to disruption or loss, particularly in older adulthood. Such losses may alter an individual’s solitude experiences, either intensifying loneliness or making space for greater self-directed solitude. This is one of many such models relevant to solitude research; we aim for SOLO to reflect construct, theories, and models exhibited in the literature. Additionally, instruments are within scope, such as the **Positive Solitude Scale** - a Likert instrument designed to measure the extent to which solitude is experienced as beneficial – and the **UCLA Loneliness Scale** – designed to measure the subjective experience of loneliness, capturing dissatisfaction with one’s social life and the frequency and intensity of perceived social disconnection.

In sum, **solitude** is a multidimensional construct encompassing both risk and resilience. Ontologically separating its subtypes - affinity, aloneliness, isolation, and withdrawal – allowing our team to clarify relationships to gerotranscendence literature and providing a foundation on which to investigate healthy aging. Table 4 displays SOLO terms and definitions discussed here.

## 4. Competency Questions Revisited

In this section, we illustrate how our preliminary ontology development can be used to provide answers to our competency questions from Table 1.

### 1. How does self-transcendence differ from gerotranscendence?

**Self-transcendence** is distinguished from **gerotranscendence** both developmentally and conceptually, given the latter’s association with themes like death acceptance and life review. These differences are encoded in the ontology through subsumption axioms and developmentally scoped realization conditions, making the distinctions machine-interpretable and suitable for reasoning.

### 2. How does the experience and impact of solitude change across the lifespan?

The SOLO ontology supports modeling lifespan changes through temporal constraints placed on the realization of the **solitude mental disposition**, especially in relation to the **late adult stage**. While **solitude** may be experienced as restorative or burdensome at any age, older adults are more likely to develop **solitude affinity**. In contrast, children and adolescents may more often exhibit **social withdrawal**.

### 3. What is solitude, and how is it distinct from social isolation, social withdrawal, and loneliness?

SOLO formalizes **solitude** as a **mental disposition** that may be realized in **processes** such as **being physically alone** or lacking social engagement, whether desired or not. **Social isolation** is defined as an objective process in which meaningful social connections are lacking. **Loneliness**, by contrast, is a **subjective affective feeling** of distress about that lack. **Social withdrawal** is a separate **mental disposition** that is habitually realized through avoidance of social interaction.

### 4. What validated instruments are used to measure gerotranscendence and solitude?

Both GERO and SOLO incorporate validated measures as instances of **Likert Scale**. For **gerotranscendence**, the **Gerotranscendence Scale** captures dimensions such as cosmic perspective, life coherence, and comfort with solitude. The **Adult Self-Transcendence Inventory** measures broader self-transcendence phenomena. SOLO includes the **Positive Solitude Scale**, capturing beneficial solitude experiences, and the **UCLA Loneliness Scale**, which measures subjective affective feelings related to **loneliness** and **social isolation**.

### 5. Is solitude necessary for developing gerotranscendence?

While not strictly necessary, **solitude** — particularly **solitude affinity** — seems critical for the realization of **gerotranscendence**. Ontologically, this can be modeled by asserting that **solitude affinity** may positively regulate the realization of **gerotranscendence**.

## 5. Discussion

The Healthy Aging through Semantic Enrichment (PHASES) provides a structured approach to understanding how solitude and gerotranscendence interact in the context of healthy aging. In this preliminary work, we have outlined progress of the PHASES project towards providing a foundation on which to represent the various ambiguous uses of solitude and gerotranscendence terminology. We are not, in this project, aiming to provide universal categories representing phenomena in these respective domains; given the current states of research in these areas, that would be premature. Rather, our initial aim is to provide ontologically precise representations of the jargon in these disciplines, in the interest of highlighting convergence and divergence among researchers. This is, as one might expect, quite challenging work. Nevertheless, our progress is promising, as we have been able to even at this early stage disentangle subtle conceptual distinctions in both gerotranscendence and solitude research sufficiently enough to address some of the competency questions that motivate our project.

While we have and will continue to make progress towards our goals, we have nevertheless met challenges. While BCIO imports BFO as its top-level, it does not use the most recent ISO standardized version of BFO. In our work, we anchored the most recent version of BCIO under the most recent version of BFO. The ISO standardized version of BFO has a richer OWL2 axiomatization, explicitly constraining domains and ranges, asserting inverses, and enforcing disjointness between the **continuant** and **occurrent** hierarchies [23], which is useful for identifying inconsistencies in ontologies. Enforcing similar constraints led to inconsistencies with BCIO when running HermiT, which we intend to raise issues for on the BCIO GitHub repository. We detail some of our concerns here.

**Figure 2:**
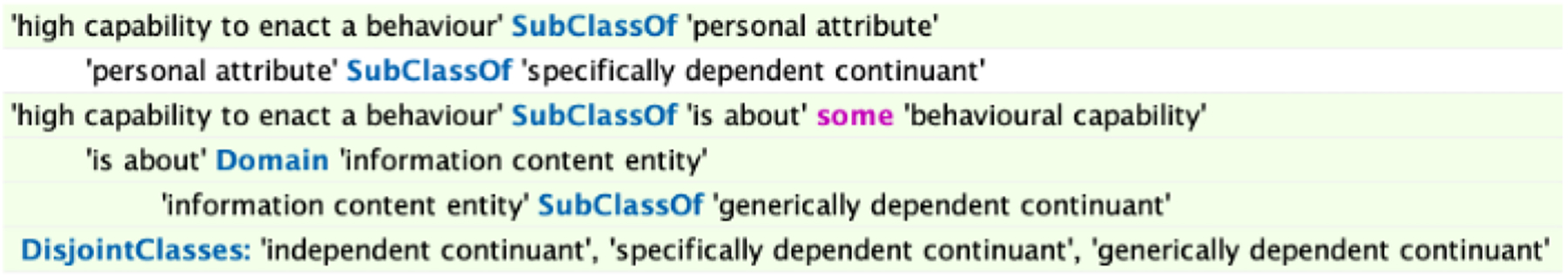
BCIO Inconsistent Use of Aboutness

In BFO, **specifically dependent continuants** are not “about” anything in the sense one finds with **information content entities**, which are defined as standing in the “is about” relationship to other entities. However, BCIO allows for **specifically dependent continuants** to be about **entities** in just this sense. Using older versions of BFO which do not enforce disjointness between **specifically dependent continuant** and **generically dependent continuant** - which is the parent class of **information content entity** – reasoners will not flag such use as inconsistent. Our anchoring of BCIO in the current version of BFO led immediately to this inconsistency, as the proof in Figure 2 displays.

**Figure 3:**
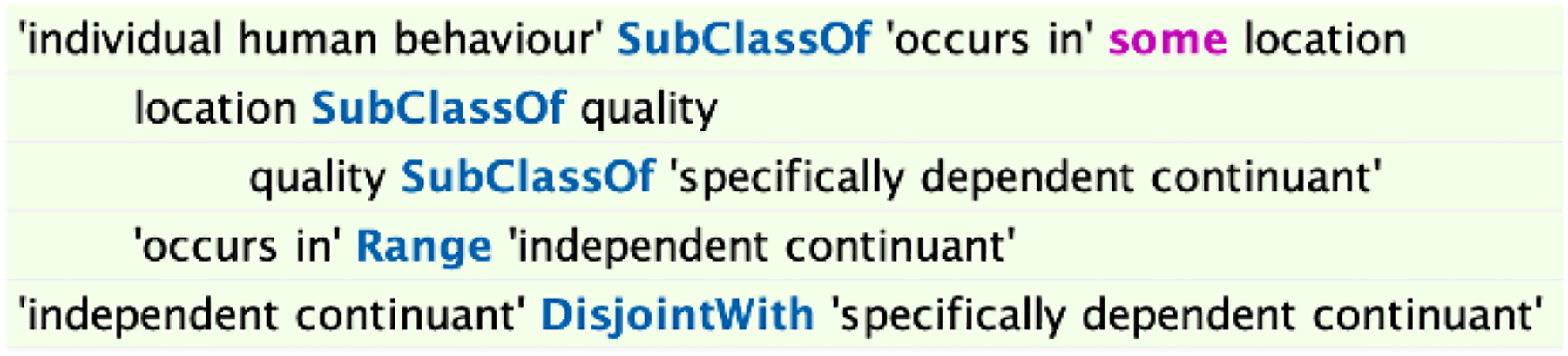
BCIO Inconsistent Use of Location

Additionally, BCIO’s defining of location as a BFO **quality** inhering in a bearer by virtue of its position relative to other entities, results in inconsistency, as displayed in Figure 3. This results in the entire subclass hierarchy of **individual human behaviour** being unsatisfiable since this class is asserted to be an **independent continuant** but inferred to be a **specifically dependent continuant**, which populates the inconsistency down the hierarchy.

## 6. Conclusion

The PHASES project (Promoting Healthy Aging through Semantic Enrichment) has developed a foundational framework for formally modeling constructs associated with solitude and gerotranscendence using ontology design best practices. By engineering two interrelated ontologies - GERO and SOLO - we provide a structured and machine-interpretable representation of concepts that have been historically ambiguous, variably defined, and inconsistently measured across the psychological and health sciences. The ontologies not only capture core terms like **self-transcendence**, **solitude**, **gerotranscendence**, and **loneliness**, but also distinguish among closely related constructs such as **social withdrawal**, **aloneliness**, and **solitude affinity**, with appropriate alignment to upper-level categories from BFO and BCIO.

A key contribution of this work lies in the formal disentangling of terminological and conceptual overlap across disciplines. The ontologies show that while many constructs share superficial similarities—e.g., solitude and social isolation, or self-transcendence and gerotranscendence—these terms differ in their realizable conditions, causal roles, and affective associations. Through a genus–species approach, supplemented by lifecycle constraints and psychometric indicators, we demonstrate how ontological modeling supports clearer construct differentiation, theory integration, and empirical operationalization.

This work has immediate utility for researchers and developers building semantically enriched knowledge systems for aging populations. GERO and SOLO make it possible to run consistent automated reasoning, validate conceptual overlaps, and bridge datasets that employ different but conceptually related labels. These ontologies also offer a template for how behavioral constructs can be modeled and integrated across disciplines using shared logical foundations. As we continue development, PHASES aims to support ontology-driven recommender and QA systems to assist gerontology researchers, clinicians, and policy stakeholders in their decision-making and evidence synthesis.

Further work is needed to incorporate cross-cultural variation, account for neurodivergent trajectories of aging, and extend interoperability with longitudinal data schemas and cohort study ontologies. We are also pursuing deeper integration with AI agents trained on domain-specific knowledge graphs, allowing for dynamic feedback between formal ontology content and emergent empirical findings. As this initiative matures, it promises to reshape how aging-related psychological constructs are theorized, measured, and applied in the service of public health and well-being.

## Figures and Tables

**Table 1:** Sample Competency Questions for GERO and SOLO Development

Competency Question
How does self-transcendence differ from gerotranscendence?
How does the experience and impact of solitude change across the lifespan?
What is solitude, and how is it distinct from social isolation, social withdrawal, and loneliness?
What validated instruments are used to measure gerotranscendence and solitude?
Is solitude necessary for developing gerotranscendence?

**Table 2:** Reused Classes and Definitions from Uber Anatomy Ontology (UBERON), Mental Functioning Ontology (MF), Behavioral Change Intervention Ontology (BCIO), NCI Thesaurus OBO Edition (NCIT), Human Phenotype Ontology (HPO), Emotion Ontology (EO), and Ontology of Biomedical Investigations (OBI)

Element	Definition
*late adult stage (UBERON)*	The lifecycle stage that is part of some post-juvenile adult stage, preceded by some prime adult stage, and involves the deterioration and loss of function over time.
*mental disposition (MF)*	A bodily disposition that is realized in a mental process.
*behaviour change intervention (BCIO)*	An intervention that has the aim of influencing human behavior.
*social isolation (BCIO)*	A mental disposition to perceive or experience oneself as isolated from and not meaningfully involved in social groups.
*temporal orientation towards the future (BCIO)*	A temporal orientation to focus more on future than present outcomes.
*Likert Scale (NCIT)*	A psychometric scale commonly used in questionnaires where a respondent is asked to evaluate an opinion according to subjective or objective criteria, by rating their level of agreement or disagreement with a statement.
*p-value (OBI)*	A quantitative confidence value that represents the probability of obtaining a result at least as extreme as that actually obtained, assuming that the actual value was the result of chance alone.
*subjective affective feeling (EO)*	An affective process that involves the experience of internal or external sensory stimuli.
*theory information content entity*	An information content entity that consists of one or more interrelated principles that purport to retrodict, explain, or predict phenomena within a circumscribed domain.
*psychoanalytic theory*	A theory information content entity that summarizes and explains mental and behavioral patterns circumscribed within the domains of society, culture, and identity.
*healthy aging*	A biological process that is realized in maintaining functional ability, emotional well-being, and social engagement in later life, relative to cultural and personal expectations.

**Table 3:** Sample GERO Classes and Definitions

Element	Definition
*transcendence*	A mental disposition borne by agents such that, if realized, is realized in shifting in goals, values, and attachments towards connections to past and future generations and in seeking coherence in one’s life narrative.
*self-transcendence*	A transcendence disposition that, if realized, is realized in shifting one’s focus beyond the self toward broader existential, moral, or spiritual concerns, including care for others, connection with nature, or alignment with perceived universal values.
*gerotranscendence*	A self-transcendence disposition borne by older adults that is shaped by the aging process and is associated with reflections on mortality and reevaluation of one’s life narrative.
*ego integrity theory*	The psychological theory developed by Erikson positing the acceptance of life as it has been lived, including acceptance of death, as the major driving psychological goal of older adults.
*reminiscence therapy*	The behaviour change intervention that uses life histories to improve well-being.
*self-transcendence theory*	The psychological theory that posits self-transcendence as a mediating role between vulnerability and well-being, influenced by personal and contextual factors.
*Gerotranscendence Scale*	The Likert scale designed by Tornstam to quantify gerotranscendence: the developmental shift in older adults marked by dimensions of cosmic transcendence, life coherence, and comfort with solitude.
*Adult Self-Transcendence Inventory*	The Likert scale designed to assess adult self-transcendence—conceptualized within wisdom literature—covering domains such as self-knowledge & integration, peace of mind, non-attachment, self-transcendence proper, and growth/presence.

**Table 4:** Sample SOLO Classes and Definitions

Terms	Definition
*solitude*	A mental disposition that, if realized, is realized in being physically alone or in the absence of social interactions, whether desired or not.
*loneliness*	A subjective affective feeling of dissatisfaction with a lack of social interactions and relationships.
*being physically alone*	A process in which an individual is physically distant from other persons or social groups, such that there is limited or no opportunity for in-person social interaction.
*social isolation state*	A process in which an individual objectively lacks social connection such that there is little or no opportunity for social interaction.
*aloneliness*	A mental disposition that, if realized, is realized in experiencing discomfort or distress due to a perceived lack of desired time alone.
*solitude affinity*	A mental disposition that, if realized, is realized in seeking out and enjoying time spent alone for reflection, creativity, or restoration.
*social withdrawal*	A mental disposition that, if realized, is realized in the consistent avoidance of social interactions with both familiar and unfamiliar peers due to psychological traits such as social fear or anxiety.
*social convoy model*	The psychological theory positing that social connections differ in levels of closeness and stability, where peripheral relationships are most vulnerable to change and termination, especially with increased age.
*Positive Solitude Scale*	The Likert scale designed to assess the degree to which individuals experience solitude as beneficial, reflecting dimensions such as emotional restoration, creativity, inner peace, and personal growth in the absence of social interaction.
*UCLA Loneliness Scale*	The Likert scale designed to assess an individual’s subjective feelings of loneliness and social isolation, as well as perceived disconnects between desired and actual social relationships.

## Footnotes

1 Both of which can be found here: https://github.com/Buffalo-Ontology-Group/phases

2 We adopt the convention of placing class and relation names in bold. Where class names are pluralized, they are to be read as referring to instances. For example, “**psychological needs**” should be read as “instances of **psychological need**”.

3 The full list of competency questions at present can be found here: https://github.com/Buffalo-Ontology-Group/phases/tree/main/documentation/competency%20questions

4 There are two issues with the UBERON definition of **late adult stage** worth highlighting. First, the parent class for **late adult stage** - UBERON_0000105 - asserts **spatiotemporal region** as its genus. In almost every case in which BFO **spatiotemporal regions** are used, **process** is more suitable; we have adjusted here. Second, **late adult stage** is given no definition in UBERON; we have supplied one here.

5 Cp. the disjunctive definition of isolation in NCIT: A state of separation; the act of isolating something; setting something apart from others; marked by separation of or from usually contiguous elements.

6 Cp. the definition of **loneliness** from the Semantic Science Integrated Ontology (SIO), which is too specific for our purposes, but which may extend from our definition: An unpleasant emotion in which a person feels a strong sense of emptiness, yearning distress and solitude resulting from inadequate quantity or quality of social relationships.

7 Cp. the Human Phenotype Ontology (HPO) definition for HP_5200110 which has alternative label “Marked Preference for Solitude”. HP_5200110 is a subclass of HPO’s **abnormal social behavior**, which makes it inappropriate for our use, given that affinity for solitude need not be abnormal.
